# An Approach for Examining the Impact of Food Group-Based Sources of Nutrients on Outcomes with Application to PUFAs and LDL in Youth with Type 1 Diabetes

**DOI:** 10.3390/nu12040941

**Published:** 2020-03-28

**Authors:** Janet A. Tooze, Natalie S. The, Jamie L. Crandell, Sarah C. Couch, Elizabeth J. Mayer-Davis, Corinna Koebnick, Angela D. Liese

**Affiliations:** 1 Department of Biostatistics and Data Science, Wake Forest School of Medicine, Winston-Salem, NC 27157, USA; 2 Department of Health Sciences, Furman University, Greenville, SC 29613, USA; Natalie.the@furman.edu; 3 School of Nursing and Department of Biostatistics, University of North Carolina at Chapel Hill, Chapel Hill, NC 27599, USA; jbigelow@email.unc.edu; 4 Department of Rehabilitation, Exercise, and Nutrition Sciences, University of Cincinnati Medical Center, Cincinnati, OH 45267-0394, USA; sarah.couch@uc.edu; 5 Departments of Nutrition and Medicine, University of North Carolina at Chapel Hill, Chapel Hill, NC 27599; mayerdav@email.unc.edu; 6 Kaiser Permanente Southern California, Department of Research & Evaluation, Pasadena, CA 91101, USA; Corinna.Koebnick@kp.org; 7 Department of Epidemiology and Biostatistics, Arnold School of Public Health, University of South Carolina, Columbia, SC 29208, USA; liese@mailbox.sc.edu

**Keywords:** dietary patterns, LDL cholesterol, polyunsaturated fatty acids, food groups

## Abstract

Traditionally, nutritional epidemiologists have utilized single nutrient or dietary pattern approaches to examine diet-health relationships. However, the former ignores that nutrients are consumed from foods within dietary patterns, and, conversely, dietary patterns may provide little information on mechanisms of action. Substitution provides a framework for estimating diet-health relationships while holding some nutrient intakes constant. We examined substitution effects of polyunsaturated fatty acids (PUFAs) in the SEARCH Nutrition Ancillary Study in the context of food group source. PUFAs were calculated from fatty acids 18:3, 20:5, and 22:6 (*n*-3), and 18:2 and 20:4 (*n*-6) from a food frequency questionnaire, quantified by food group. Models were adjusted for other fat intake, carbohydrates, protein, age, race, gender, and diabetes duration. Participants (*n* = 1441) were 14 years old on average, 51% female, with type 1 diabetes for 3.6 years. Mean intake of PUFAs was 14.9 g/day, and the highest PUFA sources were nonsolid fats, nuts, grains, red/processed meats, sweets/desserts, and high-fat chicken. PUFAs from nuts were inversely associated with low-density lipoprotein cholesterol (LDL) (*p* = 0.03) and PUFAs from high-fat chicken were positively associated with LDL (*p* < 0.01). Substituting nuts for chicken was associated with −7.4 mg/dL in LDL. These findings illustrate the importance of considering food group-based sources of nutrients when examining diet-health relationships.

## 1. Introduction

The common approach of describing the relationship between one nutrient and an outcome has identified specific mechanisms by which nutrients may affect health. For example, the intake of *n*-6 polyunsaturated fatty acids (PUFA) has been associated with lower levels of low-density lipoprotein (LDL) cholesterol and decreased risk of cardiovascular disease [1,2,3,4]. This approach, however, is a simplistic model of the impact of nutrition on health, as nutrients are typically obtained by consuming foods, which contain other nutrients and phytochemicals and are consumed within a dietary pattern. Thus, the traditional approach does not consider the specific food group-based source of a given nutrient. In addition to the nutrient-based approach, researchers have examined dietary patterns and their relationship to health. Although dietary patterns are appealing because they capture the complexity and multidimensionality of dietary intake, they provide little information in terms of potential mechanisms of action.

Substitution provides a valuable framework for both quantifying risk and exploring mechanisms of action while holding some nutrient intakes constant [5]. Using this approach, it is possible to consider nutrients and food groups simultaneously by modelling nutrients nested within food group. Only a few studies have examined the relationship between different food group sources of a nutrient and the impact on health [6,7] or examined the impact of nested food group components [8]. For example, de Oliveira Otto et al. found that a higher intake of saturated fat from dairy was associated with a significantly lower risk of cardiovascular disease (CVD), whereas a higher intake of saturated fat from meat was associated with greater risk of CVD. The authors concluded that substituting 2% of energy from meat with energy from dairy was associated with a 25% lower risk of cardiovascular disease [6]. Whereas their substitution model included energy and alcohol, it did not adjust for other fats, carbohydrates, or protein. Therefore, the specific source of the substitution on the comparison of the modelled nutrients could have been to a mix of carbohydrates, protein, and other fats, as well as one substituted saturated fatty acid for another.

The present study builds on the concept of substitution effects from a novel food group-based nutrients perspective. To illustrate our approach, we focused on the association between total PUFAs contained within food groups and LDL cholesterol using data from the SEARCH Nutrition Ancillary Study (SNAS), a study that investigated the relationship between dietary intake and complications of type 1 diabetes in youth, in a secondary data analysis. This particular application was chosen because previous research found that diets rich in PUFA, as opposed to those high in saturated fat, have been associated with lower LDL cholesterol in adults [1]. However, the relationship between PUFA intake and LDL cholesterol in youth with type 1 diabetes has been studied far less. In a small study of youth with type 1 diabetes in Australia (*n* = 79), PUFA was not associated with LDL cholesterol [9]. In contrast, among Japanese youth with type 1 diabetes, those with adolescent onset had lower levels of PUFA intake in comparison to those with childhood onset type 1 diabetes and had the highest level of LDL cholesterol [10]. We previously showed a significant inverse (favorable) association between plasma biomarkers of linoleic acid (18:2, *n*-6) and docosahexaenoic acid (22:6, *n*-3) and LDL cholesterol, and a significant positive (unfavorable) association between eicosapentaenoic acid (20:5, *n*-3) and LDL cholesterol in youth with type 1 diabetes [11]. One source of discrepancy between different estimates of the effects of PUFAs on LDL cholesterol may be the food sources of the PUFAs, i.e., different food sources contain different types of PUFAs, and examining them at the food group source level may elucidate relationships not found when examining them together. Furthermore, examining food-based sources of PUFAs may inform dietary guidance. Therefore, we sought to develop an approach to consider nutrients nested within food group sources. Specifically, we explored total PUFAs and *n*-3 and *n*-6 fatty acid subclasses separately using multivariable substitution models.

## 2. Materials and Methods

### 2.1. Study Population and Design

SEARCH for Diabetes in Youth is a population-based multi-center study of diabetes in youth less than 20 years of age with six recruitment centers (Cincinnati, Colorado, Hawaii, Seattle, South Carolina, and Southern California, including two American Indian reservation-based populations in Arizona and New Mexico directed by Colorado) [12]. Details of the study have been presented elsewhere [12,13]. Briefly, incident non-gestational cases of physician-diagnosed diabetes were identified from 2002 onward under Health Insurance Portability and Accountability Act waivers of consent. A cohort study was conducted among participants diagnosed from 2002–2005. The study was approved by the local institutional review boards at each site. The parent or guardian for those less than 18 years of age, and the participant if 18 years of age or older, provided written informed consent prior to the initiation of any study procedures or data collection, according to the requirements of the local institutional review board (IRB). The written assent of subjects who were less than 18 years of age was also governed by the requirements of the local IRB. Participants were asked to complete an initial survey, followed by an in-person visit with a physical examination, collection of blood and urine, and completion of interviewer-administered or self-administered questionnaires. This cross-sectional analysis was restricted to youth with type 1 diabetes (provider diagnosed and positive for at least one diabetes autoantibody, GAD65 or IA-2) whose diabetes was prevalent in 2001 or incident in 2002–2005 who were ≥10 years of age at the time of their baseline study visit for at least three months. 

### 2.2. Development of Food Groups

A modified Block Kids food frequency questionnaire (FFQ) with an expanded list of foods selected to reflect ethnic, cultural, and regional variation in the study population was used to assess dietary intake [14,15]. The FFQ consisted of 85 food lines for which the participant indicated if the item(s) was/were consumed in the past week (“yes/no”). If yes, participants indicated how many days and the average portion size. Portion size was queried for each line item in a manner relevant to the item either as a number (e.g., number of slices of bread) or as “very small,” “small,” “medium,” or “large” relative to pictures of food in bowls or plates provided with the form. Additional foods were captured by an open-ended question at the end of the FFQ. The FFQ was primarily self-administered after staff instruction. The recipe and portion-size databases for this instrument were modified from the respective Diabetes Prevention Program databases, using the Nutrition Data System (NDS) for Research (database 3 version 4.05/33, 2002, Nutrition Coordinating Center, University of Minnesota, Minneapolis) and industry sources [15]. The nutrient composition of the recipes was computed using Nutrition Data System for Research (NDSR 2014) developed by the Nutrition Coordinating Center, University of Minnesota, Minneapolis, MN. The SEARCH FFQ has recently been validated and shown to have reasonable characteristics for the vast majority of food groups and nutrients needed for the purpose of this analysis [15].

To develop food groups, each line item was associated with a recipe, and each ingredient of the recipe was associated with a food group. Nutrients were calculated at the ingredient level, and then summed by food group to derive food group-based sources of nutrients. Total *n*-3 plus *n*-6 PUFAs were calculated from the sum of 18:3, 20:5, and 22:6 (*n*-3), and 18:2 and 20:4 (*n*-6) as derived from the FFQ and were quantified by food group (Table 1). PUFA nutrient values were calculated for each ingredient used, but the ingredients may have been cooked or processed in some way. For example, the legume group included the ingredient of “refried beans,” which have added fats that contain PUFAs in addition to beans (Table 1). After quantifying PUFAs from ingredients by food groups, food groups that provided less than 10.5 kJ (2.5 kcal)/day from *n*-3 and *n*-6 PUFAs were collapsed into an “other” category used for modelling (sports bars, meal replacements, fruit, soy, legumes, beverages, and broths). To determine the primary food-based sources of PUFAs within each food group, the percent of PUFAs consumed from each ingredient within a food group was computed.

### 2.3. Lipid Levels

Lipid levels were assessed by the Northwest Lipid Metabolism and Diabetes Research Laboratories in Seattle, Washington from plasma samples processed at the site and shipped within 24 h. A Hitachi 917 autoanalyzer (Boehringer Mannheim Diagnostics, Indianapolis, IN) was used for assays of plasma cholesterol, triglyceride, and HDL cholesterol. The Friedewald equation was used to calculate LDL cholesterol if triglyceride concentration was <400 mg/dL (4.52 mM/L) and by the Beta Quantification procedure if triglyceride was ≥400 mg/dL [16].

### 2.4. Other Variables

Medical record abstraction was used to abstract information on clinical presentation and care. Age, gender, race, ethnicity, and date of diagnosis of diabetes were all captured by self-report. Standard census categories were used to capture race and ethnicity [17].

### 2.5. Statistical Analysis

The SNAS study was powered based on the primary aim of examining the relationship between nutrients (including PUFAs) and subclinical cardiovascular disease. For type 1 diabetes, there was over 80% power to detect a difference of 0.1 standard deviation (SD) in the continuous outcome of interest (e.g., pulse wave velocity) with a change of 1 SD in the dietary exposure. This was a secondary analysis of the SNAS study was expected have comparable power to the primary aim based on use of the same analytic method (regression of a continuous variable on nutrients of interest). Participant characteristics and intakes of PUFAs from specific food groups were summarized using descriptive statistics.

LDL cholesterol was transformed for modelling using the natural log due to skewness. Results were back-transformed to the original scale for interpretation. We fit a linear model with total PUFA intake as the predictor variable and LDL as the outcome, controlling for non-PUFA fats, protein, and carbohydrates, as well as age, race, gender, and duration of diabetes. The inclusion of all macronutrients in the model effectively adjusts for non-PUFA energy intake. Next, a model was fit including each of the different food group-based sources of total PUFA intakes (rather than their sum as in the first model) with the same covariates. This model allowed for the estimation of the relationship between PUFAs sourced from each food group with LDL. Substitution effects, i.e., the effects on LDL of trading PUFAs from one food group for another, were estimated using linear contrasts. Because the model controls for all non-PUFA macronutrients, we restrict the substitution to food group-based sources of PUFAs rather than for other macronutrients. Similar models were fit separately for *n*-3 PUFAs and *n*-6 PUFAs. Beta coefficients were reported for a 10-kcal change in the food group based PUFA to aid interpretation. All analyses were performed in SAS (v 9.4, Cary, NC), at a two-sided alpha level of 0.05.

## 3. Results

Of the 1861 participants with type 1 diabetes whose diabetes was prevalent in 2001 or incident in 2002–2005 who were ≥10 years of age at the time of their baseline study visit, 89 were excluded because they had diabetes for less than three months at the time of their study visit, 128 were missing the FFQ, 188 were excluded for missing fasting lipids, 15 were excluded for reporting energy values below 2092 kJ (500 kcal) or above 20920 kJ (5000 kcal), and six were excluded for taking lipid lowering medications, for a final analytic sample of 1435.

The participants were 14 years old on average, 51% female, and had a diagnosis of type 1 diabetes for 3.6 years, with an average energy consumption ranging from 6653 kJ/day (1590 kcal/day) to 8452 kJ/day (2020 kcal/day) characterized by age and gender, with 48% of energy from carbohydrates and 16% of energy from protein (Table 2). The mean intake of PUFAs derived from the FFQ was 14.9 g/day, or 562 kJ/day (134.4 kcal/day, 7.6% of energy), with the highest intake sources of PUFAs from nonsolid fats, nuts, grains, red and processed meats, sweets and desserts, and chicken (Table 1,Table 3). The primary sources of PUFAs by food group are presented in Table 1 (e.g., soybean oil comprised 35.9% of PUFA consumption in the nonsolid fats category). However, some food groups contributed very little to overall PUFA intake. Across all food groups, the highest food sources were soybean oil, regular ranch salad dressing, roasted peanuts, oil of unknown type, and fast food chicken nuggets.

**Figure 1:**
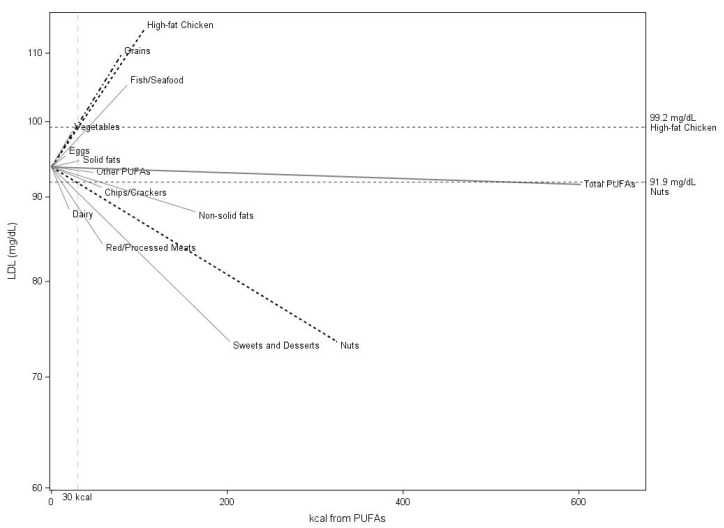
Relationship of kcal from *n*-3 and *n*-6 PUFAs by food group-based source and total PUFAs on LDL cholesterol in youth with type 1 diabetes in the in the SEARCH Nutrition Ancillary Study sample based on the model for combined food groups (Total PUFAs) and the model presented in Table 4 fit by food group, with both models adjusted for other fats, protein, carbohydrates, fiber, age, race, gender, and duration of diabetes, with back-transformation to the original scale for LDL cholesterol. The dashed black lines represent significant relationships for *n*-3 plus *n*-6 PUFAs from nuts, grains, and high-fat chicken. The length of the line represents the maximum kcal PUFA intake in the population. Solid gray lines depict other nonsignificant relationships. The dashed vertical line indicates 30 kcal/day (126 kJ/day) of *n*-3 plus *n*-6 PUFA intake, and the horizontal dashed lines indicate predicted LDL cholesterol values on the vertical axis for 30 kcal (126 kJ/day) intake of PUFAs from nuts or from chicken. See Table 1 for a definition of the food groups and predominant sources of PUFAs.

The relationship of log LDL cholesterol on *n*-3 and *n*-6 PUFAs from all food sources summed together was first examined. A significant relationship was not found (*b* = −0.0004, *p* = 0.85 for a 41.8-kJ (10-kcal) difference). Next, a model including PUFAs from the different food group-based sources was fit. In this model, PUFAs from nuts were inversely associated with the natural log of LDL cholesterol (*b* = −0.0075, *p* = 0.03 for a 41.8-kJ (10-kcal) difference) and PUFAs from high-fat chicken (*b* = 0.0182, *p* < 0.01 for a 41-8 kJ (10-kcal) difference) and grains (*b* = 0.0197, *p* = 0.05 for a 41.8-kJ (10-kcal) difference) were positively associated with LDL cholesterol; PUFAs from sweets and desserts, dairy, red and processed meats, eggs, nonsolid fats, solid fats, chips and crackers, fish and seafood, vegetables, and other sources were not significantly associated with log LDL cholesterol (Table 4) in adjusted models. As illustrated in Figure 1, slopes of the relationship between different food group-based sources of PUFAs and LDL cholesterol from this model were quite variable with some food group-based sources of PUFAs exhibiting positive relationships with LDL cholesterol (high-fat chicken, grains, fish/seafood, vegetables, solid fats, and eggs), and others exhibiting null relationships (dairy, red/processed meats, sweets and desserts, nuts, nonsolid fats, chips and crackers, and other sources of PUFAs). Most of these relationships were not statistically significant, indicating that the slopes were not statistically different from zero.

Substituting 30 kcal/day of PUFA intake from nuts for PUFA intake from high-fat chicken was associated with a decrease in LDL cholesterol of 7.4 mg/dL (95% CI: 3.4, 11.3 mg/dL, Figure 1). As indicated by the vertical dashed line in the figure, a 126 kJ/d (30 kcal/day) intake was associated with an average LDL cholesterol of 99.2 mg/dL (95% CI: 89.0, 109.5 mg/dL) when consumed from chicken, but 91.9 mg/dL (95% CI: 82.4, 101.4 mg/dL) from nuts. In addition, other fat intake was positively associated with log LDL (*p* = 0.01), and protein intake was negatively associated with log LDL (*p* < 0.01). High-fat chicken was also significantly associated with increased log LDL for *n*-3 PUFAs and *n*-6 PUFAs, and nuts were associated with decreased log LDL for *n*-6 PUFAs, when examined separately (Table S1). In the analysis of *n*-3 PUFAs, grains were positively correlated with log LDL cholesterol, and *n*-3 PUFAs from nuts were no longer statistically significant (*p* = 0.08) (Table S1). Notably, grains had a *p*-value < 0.10 for the *n*-6 PUFA model (Table S1).

## 4. Discussion

In a sample of youth with type 1 diabetes, we examined the relationship between food group-based PUFA sources and LDL cholesterol levels. The results suggest that substituting PUFAs from nuts for PUFAs from high-fat chicken would be associated with a significant decrease in LDL cholesterol. We also found that PUFA intake from grains was positively associated with LDL cholesterol.

In a pooled analysis of 25 nut intervention studies, an average consumption of 67 g of nuts per day was associated with a 10.2 mg/dL reduction in LDL [18], consistent with our analysis. A difference in the specific PUFAs and the ratios of PUFAs contained within the food groups or of other constituents of these food groups could explain differences in action of nuts compared to high-fat chicken. Nuts are high in dietary fat, ranging from 46%–76% with the exception of chestnuts, and many of them contain high levels of both PUFAs and MUFAs. Most nuts have more MUFAs than PUFAs, but the ratio varies by type of nut. For this particular FFQ, roasted peanuts were assumed to be the primary source of nuts, and fast food chicken nuggets were the primary source of high-fat chicken. Roasted peanuts consist of 49.7% fat by weight, with almost half of the fat (24.6%) from MUFA (predominantly 18:1) and almost one-third (15.7%) from PUFA, which is primarily 18:2 *n*-6 (linoleic acid) [19], and only 0.003% *n*-3 PUFAs. Chicken nuggets contain 18.4% fat by weight, with over one-third (6.8%) from monounsaturated fatty acids (MUFA, predominately 18:1), and close to one-third (6.5%) from PUFA, with the majority (7.2%) from 18:2, as well as 0.9% from 18:3 (gamma-linoleic acid) and 0.03% from 20:4 PUFAs (arachidonic acid) [19]. Both food sources predominately contained 18:2 PUFAs, suggesting it was not the type of PUFA that impacted the action.

We found only one study that had examined the relationship between grain intake and LDL cholesterol in youth with type 1 diabetes [20]. The authors identified that an increase in whole grain intake was associated with a significant decrease in LDL cholesterol, and that an increase in refined grains was associated with an increase in LDL cholesterol, although the latter did not reach statistical significance (*p* = 0.10). The cross-sectional pattern was similar, although the coefficients did not achieve statistical significance (*p*-values for both whole grains and refined grains were 0.08). As seen in Table 1, the primary PUFA sources in the grains group were from refined grain sources and are consistent with the previous study.

Although other authors have examined food and food group-specific nutrient substitution effects presented [6,7,8], this approach is not commonly used. We illustrated how the method could be used to examine the relationship between PUFAs and LDL cholesterol with careful control for other macronutrients and fiber and found differences by food group-based PUFAs that were not apparent when examining total PUFAs. A possible explanation is that aggregating the effects of PUFAs masks the effects of different food group-based sources of PUFAs. Additionally, when interpreting food group-based nutrient analyses, the food group often includes both the nutrients from the food itself (e.g., PUFAs found in chicken), as well as from preparation methods of the food (e.g., added fat). Therefore, it is important to examine the highest sources of the nutrient in a food group when drawing conclusions about food group-based effects and disseminating results.

Because foods are complex, there are multiple components that have independent and synergistic effects on health [21]. Rather than trying to isolate all these components individually, we chose to examine a plausible nutrient pathway, specifically the relationship between PUFA and LDL cholesterol, and then to examine which foods might contribute most strongly to the association between that nutrient and LDL cholesterol. It is possible that other components of the foods consumed may have beneficial or negative effects on LDL cholesterol. For example, peanuts contain higher levels of potassium, magnesium, copper, and phosphorous than chicken nuggets, and are sources of tocopherols and phenolic antioxidants that impact LDL oxidation [19,22].

One advantage of the approach used in this manuscript is that it allowed for specific dietary guidance with regards to a health outcome, e.g., substituting nuts for high-fat chicken intake lowered LDL cholesterol and held energy and total fiber intake constant in the process. It also allowed us to examine the common sources of PUFAs in this particular population of youth with diabetes to inform recommendations. Therefore, we anticipate that this approach may be useful to simultaneously examine the effects of nutrients, which offer a mechanism for the effect, and food groups, which are easily amenable to dietary guidance.

This study does have some limitations. Notably, we relied on an FFQ to estimate dietary intake. Due to its questionnaire format, several assumptions have to be made regarding recipe content and ingredients. For example, one of the FFQ line items associated with nuts were “peanuts or other nuts or seeds?”, which was assumed to primarily comprise peanuts and peanut butter, but a child’s diet may include only tree nuts or seeds and no peanuts and these differences would not be captured by the FFQ. Similarly, fast food chicken nuggets were assumed to be the most commonly consumed food for the line item “fried chicken, including chicken nuggets, chicken sandwich, or chicken wings?”, but preparation methods of fried chicken may vary widely, and nutrient quality can be affected by cooking methods. For example, frying is associated with oxidation of oils and its nutritional properties [23], and nutritional content of both chicken nuggets and peanuts has been shown to vary by cooking method [24,25]. It was also not possible to examine multiple types of food group-based sources of nutrients in the same model (e.g., saturated fat and PUFAs from nuts) due to collinearity. The use of a 24 h recall might mitigate some of these issues. Furthermore, FFQs are prone to systematic and random sources of measurement error, which may have impacted the validity of our results. However, the FFQ used in this study was developed for children as young as eight years old, and many parents assisted children under the age of 12 with completion. A validation study of this FFQ found moderate correlations for total fat (*r* = 0.40 unadjusted, 0.48 measurement error adjusted) and linoleic acid (*r* = 0.26 unadjusted, *r* = 0.37 adjusted) on the FFQ compared to 24 h recalls [16]. Furthermore, we were limited by the cross-sectional nature of the analysis.

## 5. Conclusions

Due to the complexities of diet and dietary patterns, evaluating the relationship between a nutrient and a health outcome may benefit from examining food group-based sources of nutrients, which requires quantifying nutrients within food groups from a dietary assessment tool. Relationships identified in this manner could be further tested in clinical trials or feeding study to inform dietary guidance. In particular, this analysis supports further study of the substitution of nuts for high-fat chicken intake and refined grains in youth with type 1 diabetes to lower LDL cholesterol.

## Figures and Tables

**Table 1:** Food group constituents and sources of PUFAs within food groups in youth with type 1 diabetes in the SEARCH Nutrition Ancillary Study sample.

Food Group	Constituents of Food Group	% PUFA Intakes from Food Group	Primary Sources of PUFAs in Food Group (% PUFAs in Food Group) ^1^
Nonsolid Fats	salad dressings, canola oil, corn oil, cottonseed oil, olive oil, soybean oil, vegetable oil	28.1	Soybean oil (35.9%), Ranch salad dressing regular (24.8%), unknown oil (20.3%), mayonnaise or mayo type dressing (10.1%)
Nuts	almonds, cashews, peanut butter, peanuts, walnuts	13.5	Roasted peanuts (54.7%), peanut butter (31.4%), walnuts (9.7%)
Grains	bagel, biscuit, bread, cornbread, taco shell, tortilla, buns, cereal, egg rolls, starch, French toast, grits, hush puppies, cornstarch, flour, pancakes, pita, waffles, granola, oatmeal, pasta, noodles, rice	11.3	White bread (15.7%), wheat bread (10.5%), pancakes (10.3%), flour tortilla (7.4%)
Red and Processed Meats	beef, hamburger, chili, pork, venison, bacon, bologna, corn dog, ham, hot dogs, lunch meats, sausage	8.9	Pork sausage (22.0%), hamburger (10.7%), pork ribs (9.6%), bologna (7.1%), bacon (7.0%), hot dogs (7.0%), Vienna sausage (6.6%)
High-fat Chicken	chicken nuggets (fast food), chicken with skin	7.5	Fast food chicken nuggets (69.6%), chicken (light or dark) with skin (19.4%), chicken (light or dark) with skin removed (6.9%)
Low-fat Chicken	Chicken, breast, skin removed before cooking	0.3	Chicken, breast, skin removed before cooking (100%)
Sweets and Desserts	fruit roll ups, cinnamon buns, cakes, candy, cookies, doughnuts, high fructose corn syrup, honey, ice cream, jelly, pies, pudding, sugar, syrups, turnovers	7.7	Cake (28.3%), cookies (26.9%), doughnuts (21.3%), ice cream and frozen desserts (5.7%)
Chips and Crackers	corn chips, potato chips, snack crackers, popcorn, pretzels	5.9	Snack crackers (45.0%), potato chips (24.0%), corn chips (19.1%), peanut-butter filled sandwich (5.1%)
Solid Fats	butter, shortening, animal fats, margarine, animal gravy, lard	3.3	Margarine (70.0%), vegetable shortening (15.7%), poultry gravy (5.9%), pork fat (5.9%)
Dairy	cheese, cottage cheese, whey, milk, buttermilk, cream, yogurt	3.2	Cheddar cheese (35.4%), 2% or reduced fat milk (20.4%), whole milk yogurt (13.5%), whole milk (8.7%), mozzarella cheese (5.8%)
Vegetables	broccoli, collards, salad, spinach, turnip greens, bok choy, mustard greens, parsley, spinach, Brussels sprouts, cabbage, cauliflower, relish, green beans, peppers, yeast, okra onion, squash, celery, garlic, ginger, lettuce, mushrooms, peas, potatoes, carrots, corn, BBQ sauce, catsup, salsa, tomato, tomato sauce/paste	2.4	Mashed potatoes (41.2%), corn (7.2%), green peas (5.7%)
Fish and Seafood	fish, seafood	2.3	Tuna salad with mayo (no egg) (54.1%), salmon (37.1%)
Eggs	eggs	1.8	Cooked whole eggs (52.6%), Ingredient whole eggs (29.8%), ingredient egg yolk (6.8%), boiled eggs (5.5%)
Sports Bars	granola bars, cereal bars, power bars	1.1	Cereal bars (65.5%), granola bars (31.9%)
Meal Replacements	meal replacement products, bars and drinks	1.0	Breakfast bars (74.4%), energy snack bars (14.0%), low calorie meal replacement drink (11.6%)
Fruit	apricot, bananas, figs, grapes, mango, plantains, prune, raisins, orange, tangerine, apple, applesauce, berries, cantaloupe, fruit cocktail, melon, papaya, peach, pear, persimmon, fruit juices, vinegar	0.9	Plantains (32.9%), banana (10.8%), orange juice (10.7%), apple (8.4%), apple cider (7.4%)
Soy	soy protein concentrate, soy milk, soy sauce, Worcestershire sauce, tofu, soybeans	0.5	Cooked tofu (78.4%), soy milk (18.3%)
Legumes	baked beans, refried beans, kidney beans, garbanzo beans	0.2	Refried beans (68.4%), baked beans (22.0%)

PUFA, Polyunsaturated fatty acid; ^1^ Foods indicate components of food group contributing 5% or more to the total PUFAs in the food group. Foods in bold contribute more than 5% of PUFAs overall (across all food groups). For example, soybean oil contributed to 10.1% of total PUFAs; roasted peanuts, 7.4%; ranch style dressing, 7.0%; unknown oil, 5.7%; chicken nuggets, 5.5%.

**Table 2:** Descriptive characteristics of youth with type 1 diabetes in SEARCH Nutrition Ancillary Study sample.

Characteristics	(*n* = 1435)	
	***n***	**%**
Gender		
Female	730	50.9
Male	705	49.1
Race		
White	1147	79.9
Black	103	7.2
Other	185	12.9
	**Mean**	**SD**
Age (years)	14.5	2.9
Duration of diabetes (months)	43.4	42.7
non-PUFA fats, kcal/day	508.4	241.1
Protein, kcal/day	279.7	121.2
Carbohydrates, kcal/day	839.1	356.9
Fiber, g/1000 kcal	7.9	2.4
Energy, kcal/day		
Female, 10–13 years	1613	666
Male, 10–13 years	1824	682
Female, 14–22 years	1590	633
Male, 14–22 years	2020	807

PUFA, Polyunsaturated fatty acid.

**Table 3:** PUFA nutrient intakes (kcal/day) by food group in youth with type 1 diabetes in the SEARCH Nutrition Ancillary Study sample (*n* = 1435).

	*n*-3 and *n*-6 PUFAs		*n*-3 PUFAs		*n*-6 PUFAs	
	Mean	SD	Mean	SD	Mean	SD
Nonsolid Fats	38.0	24.9	4.3	2.8	33.8	22.1
Nuts	18.3	33.3	0.4	0.9	18.0	32.5
Grains	15.3	9.7	1.3	0.8	14.0	8.8
Red and Processed Meats	11.7	9.2	1.0	0.8	10.7	8.5
Sweets and Desserts	10.5	13.9	1.1	1.4	9.4	12.6
High-fat Chicken	10.0	12.9	1.0	1.3	9.0	11.6
Chips and Crackers	8.0	7.8	0.5	0.6	7.5	7.3
Solid Fats	4.5	4.2	0.4	0.4	4.1	3.8
Dairy	4.2	2.8	1.2	1.0	3.0	1.9
Vegetables	3.2	2.9	0.6	0.6	2.6	2.5
Fish and Seafood	3.0	6.2	0.8	1.7	2.1	4.5
Eggs	2.5	2.6	0.1	0.2	2.3	2.4
Sports Bars	1.5	2.7	0.1	0.2	1.4	2.5
Meal Replacements	1.3	2.2	0.2	0.3	1.2	1.9
Fruit	1.2	1.2	0.3	0.2	1.0	1.0
Soy	0.6	2.2	0.1	0.3	0.5	1.9
Low-fat Chicken	0.4	0.7	<0.1	0.1	0.4	0.7
Legumes	0.2	0.5	0.1	0.2	0.1	0.3
Beverages and Broths	<0.1	0.1	0.0	0.0	<0.1	0.1
Other^1^	5.3	5.6	0.7	0.7	4.6	5.0
Total	134.4	67.6	13.5	6.5	121.0	61.8

PUFA, Polyunsaturated fatty acid; ^1^ Sum of food groups with total PUFA intakes < 2.5 kcal/day.

**Table 4:** Model ^1^ of *n*-3 plus *n*-6 PUFA food group-based intakes on log LDL cholesterol in youth with type 1 diabetes in the SEARCH Nutrition Ancillary Study sample (*n* = 1435).

Parameter	Estimate ^2^	SE	*p*-Value
Intercept	4.5418	0.0495	<0.0001
PUFA intakes from food group ^3^:			
Sweets/ Desserts	−0.0120	0.0080	0.1342
Grains	0.0197	0.0100	0.0495
Dairy	−0.0296	0.0415	0.4763
Nuts	−0.0075	0.0034	0.0293
Red/Processed meat	−0.0185	0.0158	0.2400
Eggs	0.0098	0.0308	0.7506
Nonsolid fats	−0.0038	0.0037	0.3069
Fats	0.0028	0.0198	0.8883
Chips/Crackers	−0.0050	0.0099	0.6156
Fish/Seafood	0.0133	0.0126	0.2913
High-fat Chicken	0.0182	0.0065	0.0053
Vegetables	0.0211	0.0301	0.4829
Other	−0.0016	0.0146	0.9127
Fats, non-PUFA	0.0042	0.0017	0.0111
Protein	−0.0078	0.0024	0.0014
Carbohydrates	0.0002	0.0004	0.5845
Total Fiber (g/1000 kcal)	−0.0031	0.0038	0.4134

^1^ Adjusted for age, race, gender, and duration of diabetes. Model includes all variables listed in table and covariates. All variables are modeled as kcal except for fiber (g/1000 kcal); ^2^ Estimate is for a 10 kcal (41.8 kJ) change (except for fiber); ^3^ See Table 1 for a definition of food groups and primary sources of PUFAs.

## Supplementary Materials

The following are available online at https://www.mdpi.com/2072-6643/12/4/941/s1, Table S1: Models^*^ of stratified n-3 and n-6 PUFA food group-based intakes on log LDL cholesterol in youth with type 1 diabetes in the SEARCH Nutrition Ancillary Study sample.
