# Supplementary material for: An Approach for Examining the Impact of Food Group-Based Sources of Nutrients on Outcomes with Application to PUFAs and LDL in Youth with Type 1 Diabetes
Source: Nutrients. 2020 Mar 28;12(4):941. doi: 10.3390/nu12040941 (PMC7230845; doi:10.3390/nu12040941)
Supplement: Supplementary file 1 [file nutrients-12-00941-s001.pdf]

Table S1. Models [1] of stratified n-3 and n-6 polyunsaturated fatty acid (PUFA) food group-based intakes on log LDL cholesterol in youth with type 1 diabetes in the SEARCH Nutrition Ancillary Study sample (n=1435)

	**n-3**			**n-6**		
**Parameter**	**Estimate [2]**	**SE**	***p*-Value**	**Estimate [2]**	**SE**	***p*-Value**
Intercept	4.5248	0.0516	<0.0001	4.5468	0.0493	<0.0001
PUFA intakes from food group [3]:						
Sweets/ Desserts	-0.0714	0.0759	0.3476	-0.0146	0.0088	0.0946
Grains	0.2451	0.1100	0.0260	0.0200	0.0109	0.0678
Dairy	0.0409	0.1046	0.6956	-0.0710	0.0615	0.2487
Nuts	-0.2161	0.1223	0.0775	-0.0082	0.0035	0.0175
Red/ Processed meat	-0.0824	0.1694	0.6268	-0.0229	0.0166	0.1684
Eggs	0.2134	0.5359	0.6905	0.0117	0.0327	0.7212
Non-solid fats	-0.0115	0.0325	0.7242	-0.0048	0.0042	0.2529
Fats	0.0020	0.1867	0.9913	0.0029	0.0222	0.8943
Chips/ Crackers	-0.1159	0.1294	0.3705	-0.0053	0.0106	0.6180
Fish/ Seafood	0.0481	0.0464	0.3000	0.0181	0.0172	0.2934
High-fat Chicken	0.1934	0.0636	0.0024	0.0194	0.0072	0.0075
Vegetables	-0.0858	0.1700	0.6140	0.0292	0.0344	0.3959
Other	0.0366	0.1303	0.7790	-0.0035	0.0162	0.8272
Fats, non-PUFA	0.0030	0.0015	0.0402	0.0045	0.0016	0.0060
Protein	-0.0066	0.0024	0.0068	-0.0078	0.0024	0.0014
Carbohydrates	0.0001	0.0004	0.7189	0.0003	0.0004	0.5193
Fiber, g/1000 kcal	-0.0021	0.0041	0.6050	-0.0032	0.0037	0.3936

[1] Adjusted for age, race, gender, and duration of diabetes. Each model (n-3 or n-6) includes all variables listed in table and covariates. All variables are expressed in kcal except for fiber (g/1000 kcal).

[2] Estimate is for a 10 kcal (41.8 kJ) change (except for fiber).

[3] See Table 1 for a definition of food groups and primary sources of PUFAs.
